# Is the pulmonary artery to aorta ratio a prognostic indicator in acute exacerbation of COPD?

**DOI:** 10.1186/s12890-025-03980-8

**Published:** 2025-11-12

**Authors:** Güzide Tomas, Ayşe Çapar, Yahya Baraç, Buğra Tollu, Cemre Abacı, Şeyma Başlılar, Bengü Şaylan

**Affiliations:** 1 İstanbul Sultan Abdülhamid Han training and research hospital, Chest Disease Clinic, İstanbul, Türkiye; 2 Respiratory İntensive Care Unit, İstanbul Sultan Abdülhamid Han training and research hospital, İstanbul, Türkiye; 3 İstanbul Sultan Abdülhamid Han training and research hospital, Radiology Clinic, İstanbul, Türkiye; 4 İstanbul Süreyyapaşa Chest disease training and research hospital, Chest Disease Clinic, İstanbul, Türkiye

## Abstract

**Introduction and aim:**

To evaluate whether the pulmonary artery to ascending aorta diameter ratio (PA/A ratio), measured via thoracic computed tomography (CT), is associated with poor clinical outcomes and mortality in patients hospitalized for acute exacerbation of chronic obstructive pulmonary disease (COPD).

**Methods:**

This retrospective study included 486 COPD patients admitted between 2017 and 2023 with available thoracic CT or CT angiography at admission. PA and aortic diameters were measured by radiologists, and PA/A ratios were calculated. Clinical parameters including blood gas values, need for non-invasive (NIMV) or invasive mechanical ventilation (IMV), and mortality data were collected. Statistical analyses included Mann-Whitney U, Chi-square, Spearman correlation, and ROC curve analysis.

**Results:**

Among the 486 patients, 451 had a PA/A ratio ≤ 1, and 35 had a PA/A ratio > 1. The median PA/A ratio was 0.80 [IQR: 0.72–0.88]. Higher PA/A ratios were significantly associated with female gender, acidosis, hypercapnia, and increased need for both NIMV and IMV (*p* < 0.05). ROC analysis identified a PA/A cut-off of > 0.80 for predicting IMV need (sensitivity 71.43%, specificity 46.02%). No significant difference was found in early or late mortality between PA/A groups. RDW and PCO₂ levels were higher in patients with PA/A > 1, while MCV was lower.

**Conclusion:**

One of the most striking findings of this study is that the PA/A ratio was not directly correlated with early or late mortality; however, it showed a significant positive correlation with the need for both non-invasive and invasive mechanical ventilation as well as presence of hypercapnia. This suggests that the PA/A ratio may be a valuable marker for predicting clinical deterioration and the need for ventilatory support rather than mortality itself.The PA/A ratio may serve as a valuable non-invasive marker in the prognostic assessment of COPD exacerbations, especially when the ratio exceeds 0.8.

**Supplementary Information:**

The online version contains supplementary material available at 10.1186/s12890-025-03980-8.

## Introduction and aim 

Chronic obstructive pulmonary disease (COPD) is characterized by systemic inflammation and recurrent exacerbations, and remains one of the leading causes of morbidity and mortality worldwide [1]. In advanced stages of the disease, hypoxemia, hypercapnia, and pulmonary vascular alterations that develop during exacerbations are among the main determinants of prognosis. These pathophysiological changes are frequently associated with the development of pulmonary hypertension, right ventricular dysfunction, and increased hospital admissions [2, 3]. As the frequency and severity of exacerbations increase, the need for invasive or non-invasive mechanical ventilation, hospital readmissions, and mortality risk rise significantly [4].

In recent years, the pulmonary artery to ascending aorta diameter ratio (PA/A ratio), measurable via chest computed tomography (CT), has gained attention as a non-invasive surrogate marker for pulmonary hypertension [5, 6]. In chronic respiratory diseases such as interstitial lung diseases and COPD, a PA/A ratio >1 has been shown to be associated with the presence of pulmonary hypertension [7, 8].

This study aimed to investigate the relationship between the PA/A ratio and adverse clinical outcomes including mortality, mechanical ventilation requirement, and gas exchange disturbances in patients hospitalized with acute exacerbation of COPD. Another aim of our study was to determine the PA/A diameter cut-off value specific to our patient population and to compare the variables that showed statistically significant associations with this value.

## Materials and methods

This retrospective study was conducted using data from hospitalized patients diagnosed with acute exacerbation of chronic obstructive pulmonary disease (AECOPD). Ethical approval was obtained from the Clinical Research Ethics Committee of Ümraniye Training and Research Hospital (Approval Date: December 12, 2023; Approval Number: B.10.1.TKH.4.34.H.GP.0.01/492). As this was a retrospective study using anonymized data, informed consent was waived by the Clinical Research Ethics Committee of Ümraniye Training and Research Hospital. Institutional permission was granted to access and utilize anonymized patient data from the hospital’s electronic medical records. All data were de-identified prior to analysis to ensure confidentiality, and the study was carried out in accordance with the principles of the Declaration of Helsinki and applicable data protection regulations. The authors declare that there are no conflicts of interest related to this study.

This retrospective study included patients over 40 years of age, who were hospitalized in the Department of Pulmonary Diseases between 2017 and 2023 due to acute exacerbation of chronic obstructive pulmonary disease (COPD) and had chest computed tomography (CT) or CT pulmonary angiography during their admission. The widest diameter of the main pulmonary artery was measured perpendicular to its long axis at the level of the bifurcation using computer-assisted calipers. The diameter of the ascending aorta was assessed from outer wall to outer wall at the level of the pulmonary artery bifurcation in the axial plane, based on non-contrast CT imaging. Representative non-contrast chest CT images demonstrating these measurements are provided as Supplementary Fig. 1.

**Fig. 1:**
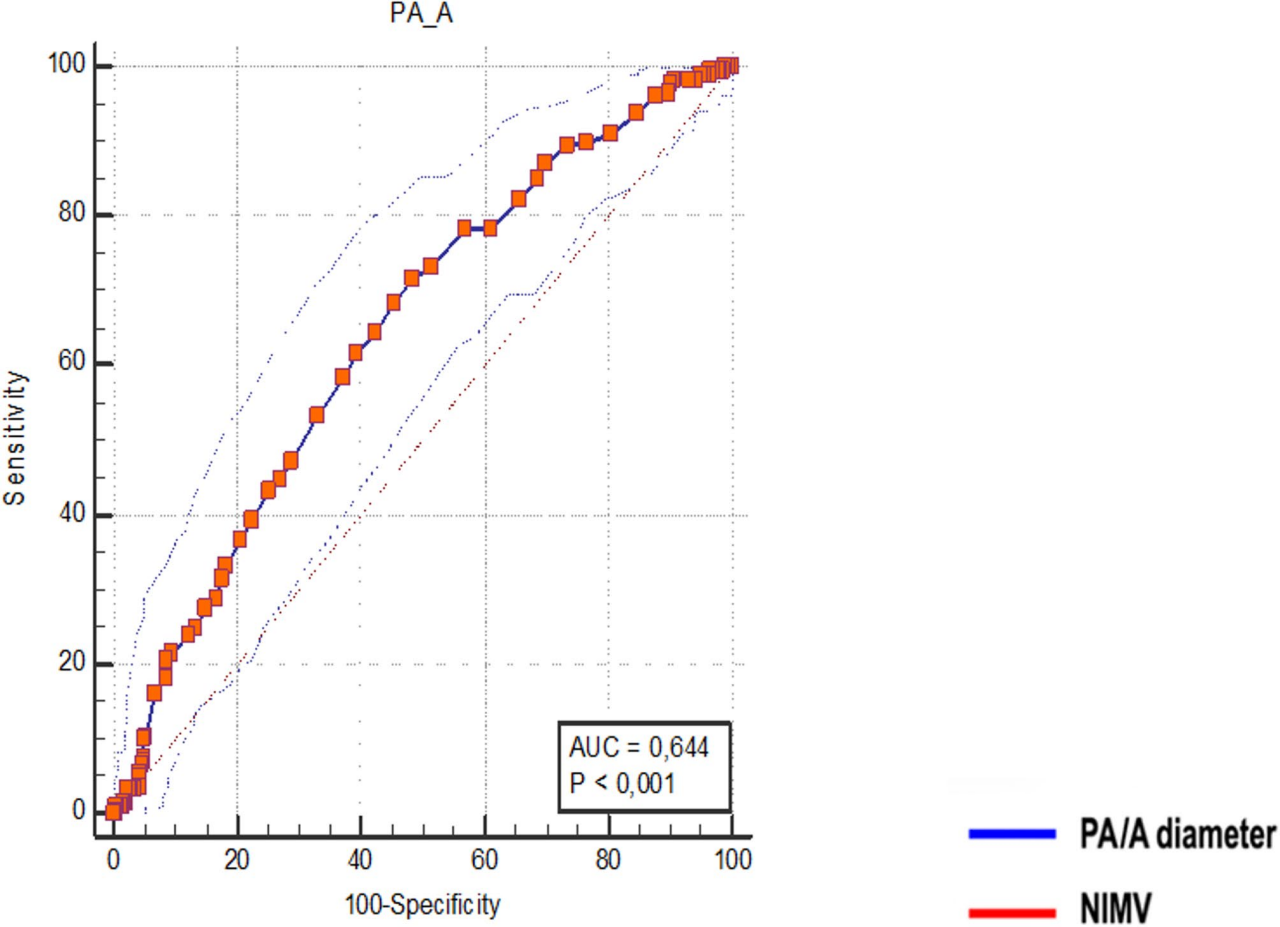
PA/A ratio, for NIMV treatment in hospitalized patients with AECOPD Note: The receiver operating characteristic (ROC) curve analysis was conducted to evaluate factors predicting in-hospital worst outcomes. Abbreviations: PA/A ratio, main pulmonary artery to ascending aorta diameter ratio; NIMV, non invazive mechanical ventilation

Group 1 (pulmonary arterial hypertension) and Group 2 (pulmonary hypertension due to left heart disease) were not included, nor were patients with obstructive sleep apnea. Patients with CTEPH, ILD, cystic fibrosis, and bronchiectasis were excluded to maintain a more homogeneous cohort of COPD exacerbations, as these conditions may independently influence both pulmonary artery/aortic diameters and clinical outcomes.Patients were excluded if image quality was insufficient for evaluation due to artifacts, or if they had a diagnosis of chronic pulmonary thromboembolism, cystic fibrosis, bronchiectasis, diffuse interstitial lung disease, or lung cancer.

PA and A diameters were measured by a radiologist who was blind to the study. Laboratory data (complete blood count, D-dimer, arterial blood gas, pro- BNP, troponin) obtained at the time of admission, mortality status, and the need for non-invasive or invasive mechanical ventilation and pulmonary artery pressure on echocardiography (PAP) in patients who were examined were recorded. These variables were compared with the PA/A ratio. The calculated PA/A cut-off value specific to our patient population, as well as the threshold of > 1, were both used to compare associated clinical and laboratory parameters.Additionally, mortality data were categorized as early mortality (at hospital) and late mortality (within 6 months), and their relationship with the PA/A ratio was evaluated.

### Statistical analysis

Data were analyzed using SPSS version 15.0 for Windows. The Kolmogorov-Smirnov test was used to assess the normality of numerical variables. Descriptive statistics were presented as number and percentage for categorical variables, and as median and interquartile range (IQR) for continuous variables due to non-normal distribution. The Mann-Whitney U test was used to compare continuous variables between groups. The Chi-square test was applied to compare categorical variables. Correlations between numerical variables were evaluated using Spearman’s correlation analysis due to non-parametric data distribution. Receiver Operating Characteristic (ROC) curve analysis was performed to determine cut-off values. A p-value of < 0.05 was considered statistically significant.

## Results

540 patients hospitalized during study period were evaluated. After applying the exclusion criteria, 486 patients were included in the final analysis. Among these, 451 patients had a PA/A ratio ≤ 1, and 35 patients had a PA/A ratio > 1.

The median PA/A ratio was calculated as 0.80 [IQR: 0.72–0.88]. Analyses were conducted using both 0.80 and 1.00 as cut-off values. There was no statistically significant difference in the rates of current or former smoking or total tobacco consumption between males and females. However, the proportion of never-smokers was significantly higher in males compared to females (*p* = 0.022). (Table 1).

**Table 1:** Demographic, clinical, laboratory data and Echocardiographic and radiological parameters of AECOPD population (n% or median(IQR))

**N=486**		
Age (years)		73 [65–81]
Sex	Male	312 (64,2)
	Female	174 (35,8)
Active smoking		132 (27,6)
Ex-smoker		343 (71,8)
Nonsmoker		46 (9,6)
Amount of cigarettes		50 [35–65]
Biomass exposure		50 (10,3)
Comorbidities		242 (49,8)
Choronic liver disease		92 (18,9)
Choronic renal disease		91 (18,7)
Diabetes Mellitus		120 (24,7)
Troponin-I (ng/mL)		12 [6,3–28]
Troponin-t (ng/mL)		19 [11,4–40,7]
BNP (pg/mL)		519 [138-17]
Lactate (mmol/L)		1,65 [1,24-2,28]
D-dimer (µg/ml)		1 [0,5-2,07]
Hemoglobin (g/dl)		12,5 [10,9–14,1]
Hematocrit (%)		38,7 [34,4–43,14]
RDW (%)		14,5 [13,5–15,8]
MCV (%)		88,6 [84,6–92]
Neutrophil (%)		7,59 [5,47-10,3]
C-Reaktive Protein (mg/dl)		30,24 [8,67–82,5]
PaCO2 (mmHg)		48,8 [42,6–58,65]
pH		7,38 [7,34-7,43]
PaO2 (mmHg)		61,1 [48,2–80]
SO2 (%)		91,5 [82,4–96,7]
Acidosis		146 (30,0)
Hypercapnia		320 (65,8)
Hypoxia		207 (42,6)
PA diameter (mm)		29 26–32 []
Aorta diameter (mm)		36 [34–39]
PA/A ration Median		0,80 [0,72-0,88]
PA/A ration (%)	1≥	451 (92,8)
	>1	35 (7,2)
Echocardiography (PAP) (mmHg)		35 [30–50]

*Abbreviations*: *AECOPD*, acute exacerbations of chronic obstructive pulmonary disease; *PA/A*, main pulmonary artery to ascending aorta diameter ratio; *RDW*, blood red cell distribution width; *BNP*, brain natriuretic peptide; *PA*, main pulmonary artery; MCV mean corpuscular volüme;PAP, pulmonary artery pressure

The PA/A ratio was found to be significantly higher in female patients, and in those with acidosis, hypercapnia, and who required non-invasive (NIMV) or invasive mechanical ventilation (IMV) compared to those without these conditions (*p* < 0.001, *p* = 0.001, *p* < 0.001, *p* < 0.001, and *p* = 0.012, respectively). (Table 2)

**Table 2:** Clinical outcomes: mortality, RICU and ventilator support (n% or median(IQR))

NIMV	180 (37,0)
IMV	84 (17,3)
RICU follow up	92 (18,9)
RICU stay duration (days)	7,5 [4,25 − 12,75]
Ward hospitalisation (days)	6 [3,9–9,5]
One-year mortality	35 (7,2)
Mortality after 1 year	87 (17,9)

*Abbreviations*: *RICU*, respiratory intensive care units; *IMV*, invaziv mechanical ventilation; *NIMV*; non-invazive mechanical ventilation

The PA/A ratio showed a weak but statistically significant positive correlation with proBNP, pulmonary artery pressure on echocardiography (PAP), RDW, and PaCO₂ levels, and a weak negative correlation with blood pH level (*p* = 0.013, *p* = 0.025, *p* < 0.001, *p* < 0.001, and *p* < 0.001, respectively). (Table 3)

**Table 3:** Correlation analysis between PA/A ratio and clinical/laboratory parameters

	PA/A	
	r	p
**Age (years)**	−0,068	0,137
**Amount of cigarettes**	0,043	0,464
**Troponin-I (**ng/mL)	−0,065	0,353
**Troponin-t (**ng/mL)	0,033	0,526
**BNP (**pg/mL)	0,126	**0**,**013**
**Lactate (**mmol/L)	−0,047	0,330
**D-dimer** (µg/ml)	0,006	0,904
**ECHO PAP** (mmHg)	0,165	**0**,**025**
**Hemoglobin** (g/dl)	−0,020	0,673
**Hematocrit** (%)	0,028	0,546
**RDW** (%)	0,163	**< 0**,**001**
**MCV** (%)	−0,057	0,219
**Neutrophil** (%)	0,007	0,880
**C-reaktive protein** (mg/dl)	−0,033	0,473
**PaCO2 (**mmHg)	0,249	**< 0**,**001**
**pH**	−0,165	**< 0**,**001**
**PaO2 (**mmHg)	0,068	0,136
**SO2** (%)	0,030	0,516
**Ward stay (**days)	0,080	0,078
**RICU stay(**days)	−0,022	0,862

Bold represents statistically significant

*Abbreviations*: *BNP*, brain natriuretic peptide; *RICU*, respiratory intensive care units; *RDW*, red blood cell; *MCV*, mean corpuscular volüme; *ECHO-PAP*, echocardiographi pulmonary artery pressure; *PA/A*, main pulmonary artery to ascending aorta diameter ratio

When PA/A > 0.80 was taken as the cut-off value, there was a statistically significant increase in the need for both IMV and non -IMV. Similarly, hypercapnia and acidosis were also significantly more common above the 0.80 cut-off point (figüre 1 and 2).

**Fig. 2:**
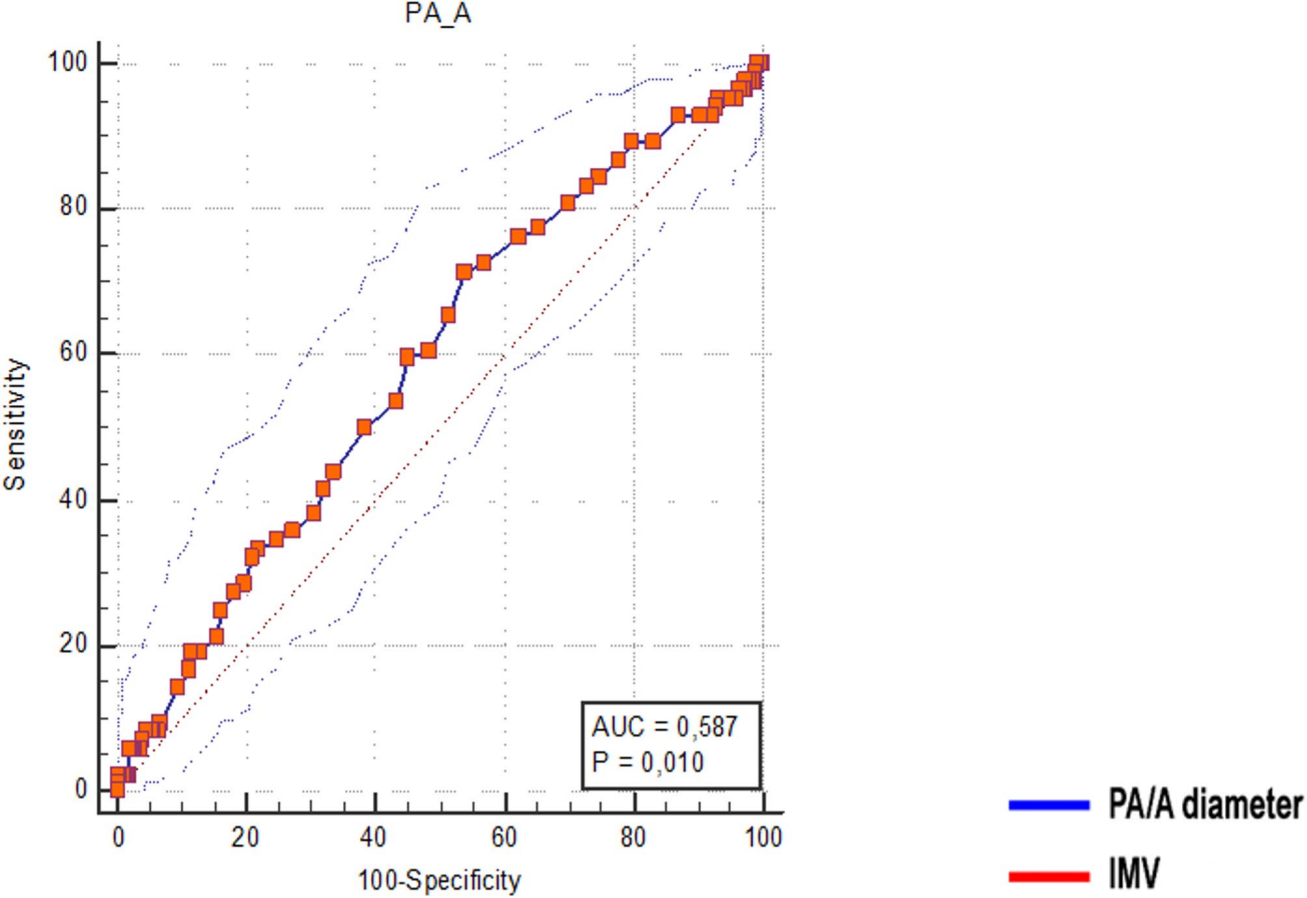
PA/A ratio, for IMV treatment in hospitalized patients with AECOPD Note: The receiver operating characteristic (ROC) curve analysis was conducted to evaluate factors predicting in-hospital worst outcomes. Abbreviations: PA/A ratio, main pulmonary artery to ascending aorta diameter ratio; IMV, invazive mechanical ventilation

The PA/A cut-off value for predicting IMV requirement was determined as > 0.80, with 71.43% sensitivity and 46.02% specificity. (Table 4).

**Table 4:** Comparison of PA/A ratio by clinical and demographic subgroups

		PA/A		
		Median	IQR	
**Sex**	Male	0,78	0,70 − 0,86	**< 0**,**001**
	Female	0,83	0,75 − 0,94	
**Smoking status**	None-smoker	0,80	0,72 − 0,88	0,809
	Smoker	0,80	0,71 − 0,90	
**Biomass exposure**	No	0,79	0,72 − 0,88	0,168
	Yes	0,82	0,75 − 0,92	
**Comorbidities**	No	0,80	0,72 − 0,89	0,885
	Yes	0,80	0,72 − 0,87	
**Chronic liver disease**	No	0,80	0,72 − 0,89	0,909
	Yes	0,80	0,72 − 0,87	
**Chronic renal disease**	No	0,80	0,72 − 0,88	0,833
	Yes	0,80	0,73 − 0,88	
**Diabetes mellitus**	No	0,80	0,73 − 0,88	0,152
	Yes	0,78	0,70 − 0,86	
**Acidosis**	No	0,79	0,71 − 0,87	**0**,**001**
	Yes	0,82	0,75 − 0,93	
**Hypercapnia**	No	0,77	0,69 − 0,84	**< 0**,**001**
	Yes	0,81	0,74 − 0,91	
**Hypoxia**	No	0,70	0,72 − 0,80	0,306
	Yes	0,72	0,79 − 0,87	
**NIMV**	No	0,77	0,70 − 0,86	**< 0**,**001**
	Yes	0,83	0,76 − 0,93	
**IMV**	No	0,79	0,71 − 0,87	**0**,**012**
	Yes	0,82	0,76 − 0,93	
**RICU follow up**	No	0,79	0,71 − 0,88	0,075
	Yes	0,82	0,74 − 0,92	
**One year mortality**	No	0,80	0,72 − 0,88	0,182
	Yes	0,77	0,66 − 0,86	
**Mortality after 1 year**	No	0,80	0,72 − 0,88	0,876
	Yes	0,79	0,72 − 0,88	

Bold represents statistically significant

*Abbreviations*: *RICU*, respiratory intensive care units; *IMV*, invaziv mechanical ventilation; *NIMV*; non-invazive mechanical ventilation; *PA/A*, main pulmonary artery to ascending aorta diameter ratio

Patients with PA/A > 1 were significantly more likely to be female, have a history of active smoking, acidosis, hypercapnia, and require NIMV compared to those with PA/A ≤ 1 (*p* = 0.023, *p* = 0.018, *p* = 0.018, *p* = 0.036, *p* = 0.003, *p* = 0.028, and *p* = 0.002, respectively). However, no statistically significant difference was observed between the groups in terms of early and late mortality rates.(Table 5).

**Table 5:** Comparison of early and late mortality based on PA/A Cut-off value of 0,80

	**PA/A**		
	≤ 0,80	> 0,80	p
Early Mortality n (%)	18 (8,6)	17 (6,1)	0,296
Late Mortality n (%)	35 (16,7)	52 (18,8)	0,564

*Abbreviations*: *PA/A*, main pulmonary artery to ascending aorta diameter ratio

In patients with PA/A > 1, RDW and PCO₂ levels were significantly higher, while MCV values were significantly lower compared to those with PA/A ≤ 1 (*p* = 0.031, *p* = 0.006, and *p* = 0.015, respectively).(Table 6).

**Table 6:** Comparison of Cardiac, Laboratory, and blood gas parameters between patients with PA/A Ratio ≤ 1 and > 1

	PA/A		
	1 ve altı	> 1	
	**Median [IQR]**	**Median [IQR]**	p
**Troponin-I (**ng/mL)	12,05 [7–28]	8 [3–30]	0,173
**Troponin-t** (ng/mL)	19 [11,31–41]	15,45 [11,55 − 28,09]	0,474
**BNP (**pg/mL)	519 [134–1695]	594,5 [196,7–2445,5]	0,388
**Lactate (**mmol/L)	1,69 [1,27 − 2,3]	1,6 [1,02–2]	0,246
**D-dimer** (µg/ml)	1,05 [0,5 − 2,11]	0,675 [0,5 − 1,93]	0,271
**ECHO(PAB) (**mmHg)	35 [30–50]	35 [25–52,5]	0,983
**Hemoglobin(g/dl)**	12,5 [10,9–14,2]	11,9 [10–13,5]	0,122
**Hematocrit**(%)	38,8 [34,6–43,3]	37,4 [32,5–41,1]	0,251
**RDW (%)**	14,4 [13,5–15,8]	15,3 [14–16,4]	**0**,**031**
**MCV(%)**	88,8 [85–92]	84,7 [78–90,7]	**0**,**015**
**Neutrophil** (%)	7,58 [5,44 − 10,31]	7,94 [5,85 − 9,18]	0,955
**C-reaktive protein** (mg/dl)	30,2 [8,5–83,8]	29,8 [13,7–47,9]	0,828
**PaaCO2**(mmHg)	48,2 [42–58,1]	55,7 [48–63,5]	**0**,**006**
**pH**	7,38 [7,34 − 7,43]	7,37 [7,31 − 7,44]	0,458
**PaO2**(mmHg)	60,9 [47,9–80]	70,1 [51,3–80]	0,413
**SO2** (%)	91,2 [82,4–96,7]	93,1 [82–95,9]	0,734

Bold represents statistically significant

*Abbreviations*: *BNP*, brain natriuretic peptide; *RDW*, red blood cell; *MCV*, mean corpuscular volüme; *ECHO-PAP*, echocardiographi pulmonary artery pressure; *PA/A*, main pulmonary artery to ascending aorta diameter ratio

## Discussion and conclusion

One of the most striking findings of this study is that the PA/A ratio was not directly correlated with early or late mortality; however, it showed a significant positive correlation with the need for both non-invasive and invasive mechanical ventilation as well as presence of hypercapnia. This suggests that the PA/A ratio may be a valuable marker for predicting clinical deterioration and the need for ventilatory support rather than mortality itself.

Pulmonary vascular remodeling and the resulting pulmonary hypertension, frequently observed in advanced COPD, are significant causes of morbidity and adverse outcomes, including reduced survival [9, 10]. Therefore, evaluating the pulmonary circulation—particularly by non-invasive methods—is critically important for prognostication in patients experiencing acute exacerbations of COPD.

Although the clinical utility of the PA/A ratio has been more clearly defined in interstitial lung diseases, its prognostic significance in patients hospitalized with acute exacerbation of COPD remains uncertain. Specifically, evaluating its association with mortality, the need for mechanical ventilation, and gas exchange abnormalities may provide evidence to support the use of the PA/A ratio as a practical prognostic biomarker in clinical settings.

In previous studies it was reported that the one year [5] and ICU mortality rates [6] were increased among AECOPD patients with a PA/A ratio >1. However we failed to show a relation between PA/A ratio which may be according to small number of patients who had increased PA/A ratio but we suggest that PA/A ratio >1 may be related to poor clinical course i.e. need for ICU follow up and NIMV/IMV need. So this parameter may be useful in.

predicting critically ill patients who should be closely followed up.and may be a useful in clinical decision-making processes.

First, due to its retrospective and single-center design, there is a risk of selection bias and unmeasured confounding factors, which may limit the generalizability of the findings. The inability to control for all potential clinical variables and disease severity indices may affect the robustness of the associations observed between the PA/A ratio and adverse clinical outcomes.

Furthermore, the precise cut-off value for PA/A ratio is not clear yet. Some of the studies were performed with a cut-off value of 0.8 and 0.9 [11]. Rho at all found that; Receiver operating characteristic analysis of these variables indicates that they may serve as a good predictive value for severe exacerbation (area under the curve, 0.77–0.78). The range of cut-off value for PA: AA ratio was 0.8 to 0.87 [11]. So we also calculated a cut-off value for our study group which was 0.8 and reevaluated whether this value may be used for prediciting mortality. In the patients who had PA/A >1(*n* = 35), RDW levels were significantly higher. This finding is consistent with the study by Cheng et al. [7]. Elevated RDW has previously been associated with cardiac stress and systemic inflammation, as well as with prolonged hospital stay and poor prognosis [7, 8]. A similar trend was observed in our study.

Interestingly, a positive correlation was found between the PA/A ratio and the amount of cigarette consumption. This finding may reflect the impact of smoking on both airway and vascular structures in COPD patients.

In light of these findings, *Our findings suggest that the PA/A ratio could potentially serve as a useful marker in clinical practice; however*,* further prospective studies are needed before recommending its routine use during thoracic CT evaluations in COPD exacerbations.* Particularly in patients with a PA/A ratio greater than 0.8, the need for ventilatory support and intensive care should be more carefully assessed. Nevertheless, to establish clinically applicable cut-off values for the PA/A ratio, larger, multicenter, and prospective studies are required.

## Limitations

This study has several important limitations that should be considered when interpreting the results.First, due to its retrospective and single-center design, there is a risk of selection bias and unmeasured confounding factors, which may limit the generalizability of the findings. The inability to control for all potential clinical variables and disease severity indices may affect the robustness of the associations observed between the PA/A ratio and adverse clinical outcomes.

Second, the measurement of the pulmonary artery and ascending aortic diameters was performed manually by a single radiologist using thoracic computed tomography at a single time point during admission. While this approach ensures consistency, the absence of inter- or intra-observer variability assessment limits the reproducibility and external validity of the PA/A ratio. Automated or blinded multi-observer assessments could have provided a more objective measurement framework.

Third, although the PA/A ratio has been proposed as a surrogate marker for pulmonary hypertension, the study did not include confirmatory diagnostic tools such as right heart catheterization or advanced echocardiographic parameters (e.g., tricuspid regurgitation velocity, right ventricular function indices). This limits the ability to validate the radiological findings with hemodynamic gold standards. Another limitation of our study is that detailed data on COPD heterogeneity, such as GOLD staging and frequent exacerbator status, were not available for all patients. This may have limited the interpretation of our findings. Additionally, follow-up data regarding long-term outcomes after discharge (e.g., 30-day or 90-day mortality, rehospitalization) were not available, which restricts the assessment of the PA/A ratio’s predictive value beyond the acute phase.

Future prospective, multicenter studies incorporating comprehensive echocardiographic and hemodynamic evaluation are warranted to confirm the prognostic value and clinical applicability of PA/A ratio in acute exacerbations of COPD.

## Supplementary Information

Supplementary Material 1

## Data Availability

The datasets used and/or analyzed during the current study are available from the corresponding author on reasonable request.
