# Supplementary material for: Is the pulmonary artery to aorta ratio a prognostic indicator in acute exacerbation of COPD?
Source: BMC Pulm Med. 2025 Nov 12;25:522. doi: 10.1186/s12890-025-03980-8 (PMC12613874; doi:10.1186/s12890-025-03980-8)

sThe widest diameter of the main pulmonary artery (30.06 mm), measured perpendicular to its long axis at the level of the PA bifurcation, was determined using computer-assisted calipers.


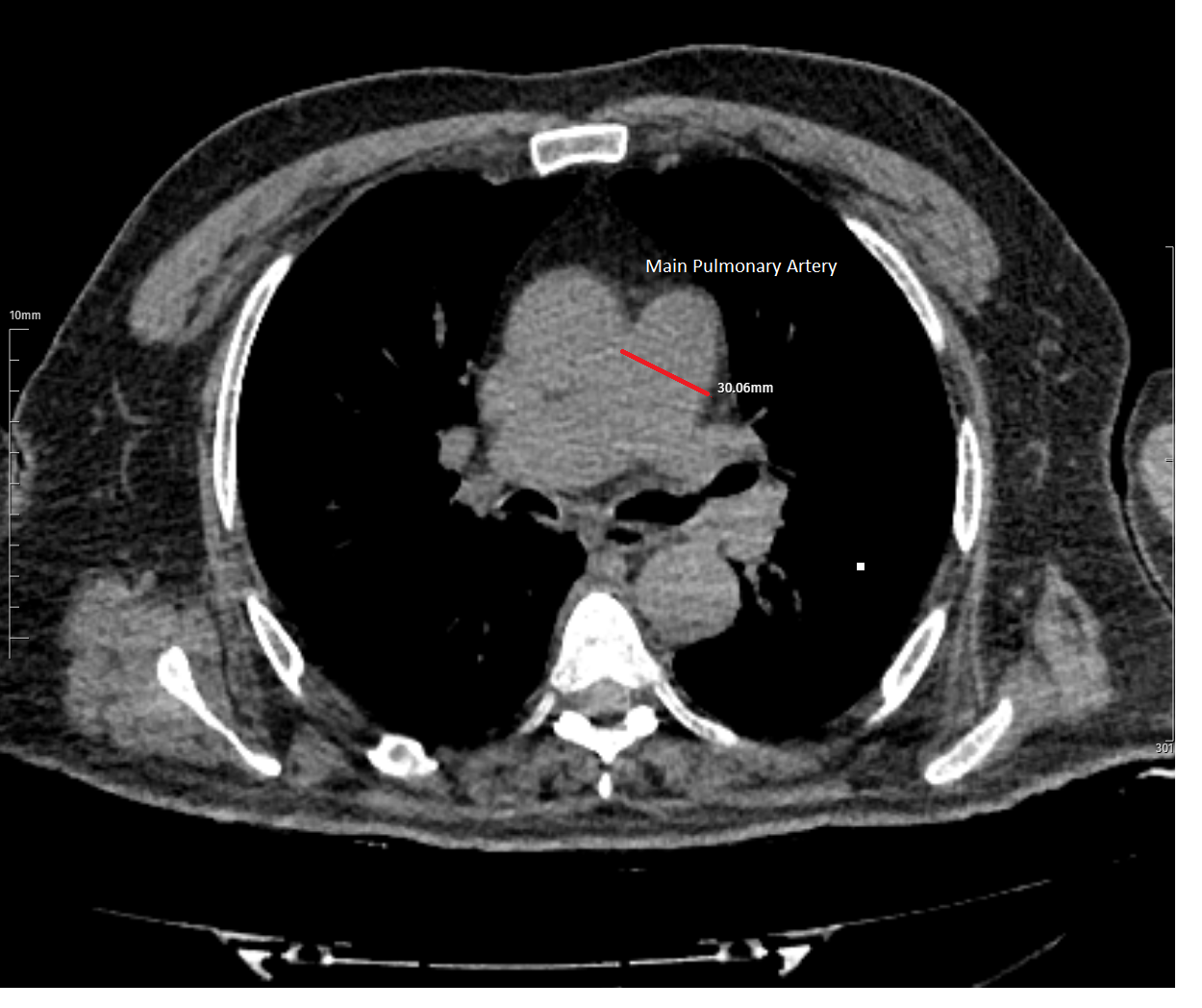


The diameter of the ascending aorta(39.46 mm), measured from the outer wall of the aorta at the level of the pulmonary artery bifurcation in the axial plane, was assessed using non-contrast CT imaging


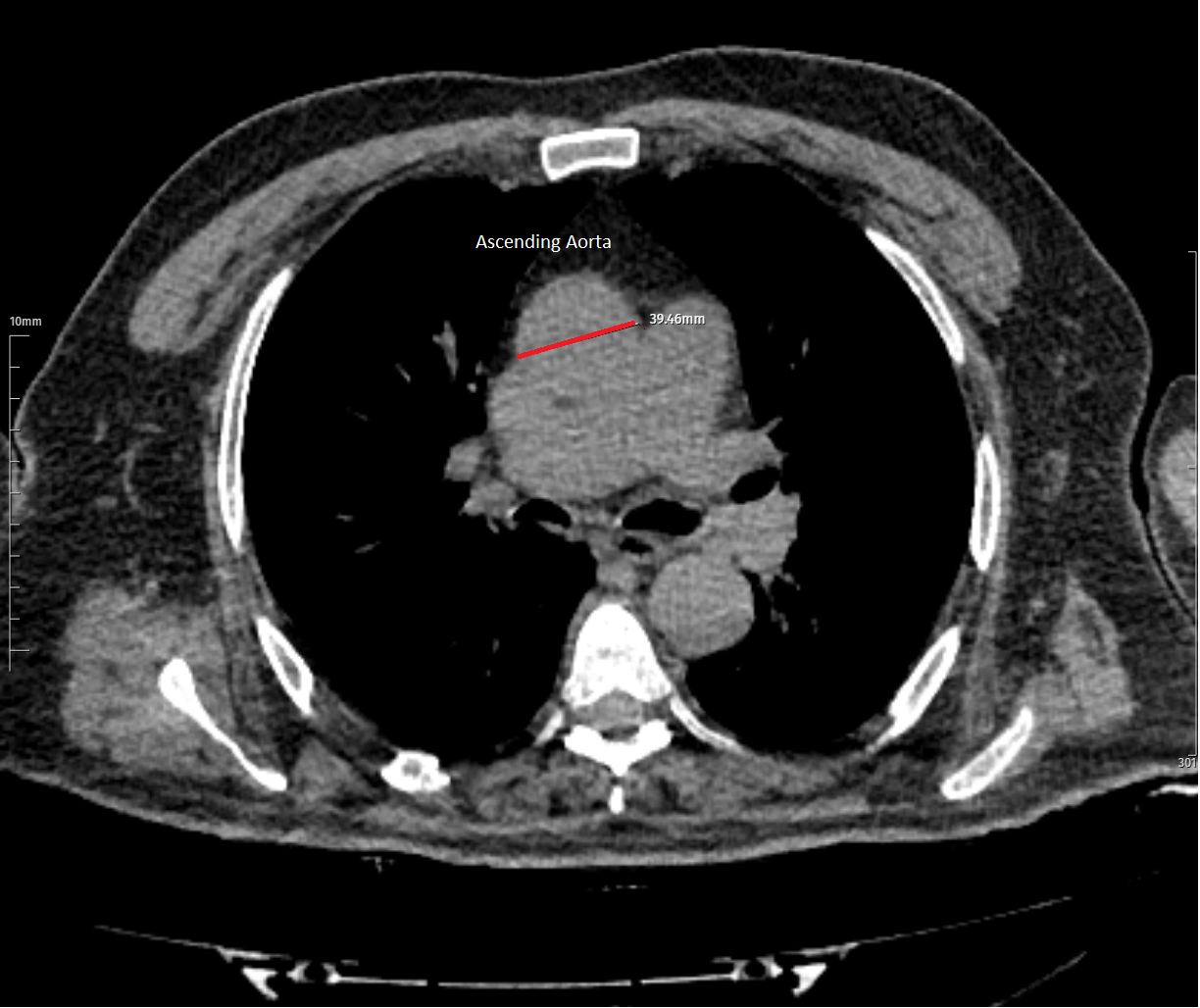

Supplement: Supplementary file 1 — Supplementary Material 1 [file 12890_2025_3980_MOESM1_ESM.docx]
